# Current management of upper lid ptosis: a web-based international survey of oculoplastic surgeons

**DOI:** 10.5935/0004-2749.2021-0105

**Published:** 2023

**Authors:** Alicia Galindo-Ferreiro, Denise C. M. Zornoff, Jose Eduardo Correntes, Patricia M. Akaishi, Silvana A Schellini

**Affiliations:** 1 Department of Ophthalmology, Rio Hortega University Hospital, Valladolid, Spain; 2 Distance Education and Health Information Technology Center, School of Medicine of State University of São Paulo, Brazil; 3 Department of Biostatistics, Biosciences Institute, Universidade de São Paulo, SP, Brazil; 4 Department of Ophthalmology, Faculdade de Medicina de Ribeirão Preto, Universidade de São Paulo, SP, Brazil; 5 Department of Ophthalmology, Faculdade de Medicina, Universidade Estadual Paulista “Júlio de Mesquita Filho”, Botucatu, SP, Brazil

**Keywords:** Blepharoptosis/diagnosis, Amblyopia, Phenylephrine, Surveys and Questionnaires, Demography, Surgeons, Blefaroptose/diagnóstico, Ambliopia, Fenillefrina, Inquéritos e questionários, Demografia, Cirurgiões

## Abstract

**Purpose:**

This study aimed to evaluate the current practice patterns for assessing and managing upper lid ptosis among members of the Latin American and Spanish societies of Ophthalmic Plastic and Reconstructive Surgery.

**Methods:**

An e-mail was sent to invite members of both societies to participate in this anonymous web-based survey. The survey collected data on surgeons’ demographics and four other sections: upper lid ptosis preoperative evaluation, surgical preferences, postoperative management, and complications. The frequency and proportions of the responses were then statistically analyzed.

**Results:**

The survey was responded by 354 experienced oculoplastic surgeons, 47.7% of whom generally performed more than 20 upper lid ptosis surgeries annually. Of those respondents, 244 (68.9%) routinely check for dry eye preoperatively. Less than half of the respondents (47.4%) perform the phenylephrine test for congenital or acquired ptosis. Mild upper lid ptosis was reported to be usually corrected with conjunctival mullerectomy (43.6%). Severe upper lid ptosis was reported to be usually corrected with frontalis surgery (57%), followed by anterior levator resection, mainly supramaximal resection (17.5%). In cases of severe congenital ptosis, the main reason for surgery was to alleviate the risk of amblyopia (37.3%). An anterior approach was reported to be usually (63.3%) used to manage involutional ptosis associated with dermatochalasis. Common complications comprised undercorrection after levator resection (40%) or frontalis suspension (27.5%).

**Conclusions:**

This study reports the current practice patterns among Spanish and Latin American oculoplastic surgeons in upper lid ptosis diagnosis and treatment. Surgeons can use this study data to compare disease management with their colleagues.

## Abstract (pt)

**Objetivo:**

Avaliar a prática e tratamento da ptose da pálpebra superior por membros das sociedades latino-ame­ricanas e espanhola de Cirurgia Plástica Ocular.

**Métodos:**

Os membros das referidas sociedades foram convidados por e-mail para responder a um questionário eletrônico garantindo o anonimato. O questionário constou de dados demográficos do cirurgião e outras quatro seções: avaliação pré-operatória da ptose da pálpebra superior, preferências cirúrgicas, conduta pós-operatória e complicações. Estatística descritiva foi utilizada para análise da frequência e proporções percentuais.

**Resultados:**

Trezentos e cinquenta e quatro experientes cirurgiões oculoplásticos dos quais 47,7% realizam mais de 20 cirurgias de ptose da pálpebra superior por ano responderam ao questionário. Na avaliação pré-operatória, 68,9% realizam testes para olho seco, mas o teste da fenilefrina é feito por menos da metade dos entrevistados (47,4%). A ptose da pálpebra superior leve geralmente é corrigida por conjuntivo-mullerectomia (43,6%), a ptose da pálpebra superior grave por cirurgia do músculo frontal (57%) ou ressecção da aponeurose do levantador via anterior, principalmente usando a supramáxima (17,5%). O principal motivo para operar a ptose congênita grave é o risco de ambliopia (37,3%). A ptose involucional associada à dermatocálase costuma ser corrigida pela via anterior (63,3%). Hipocorreção é complicação comum após a ressecção da aponeurose do levantador (40%) ou suspensão ao frontal (27,5%).

**Conclusões:**

As práticas atuais dos cirurgiões oculoplásticos espanhóis e latino-americanos para diagnóstico e tratamento de ptose da pálpebra superior foram relatadas. Os dados apresentados podem ser usados para comparar a abordagem dos cirurgiões com a de seus pares.

## INTRODUCTION

Upper lid ptosis (ULP) is a frequent lid malposition with several causes. Furthermore, ULP management comprises diverse approaches. Most publications on ptosis are case series, reporting different surgical techniques and their outcomes. However, currently, there is no consensus on the optimal preoperative evaluation, surgery type, and postoperative strategy to manage ULP.

There are two web-based surveys on ULP surgical management. The first was published in 2011 by the American Society of Ophthalmic Plastic and Reconstructive Surgeons (ASOPRS) to identify trends in ptosis management^(1)^, and the other was published in 2016 by the British Oculoplastic Surgery Society (BOPSS) to evaluate patient satisfaction^(2)^.

Currently, there have been no publications on the ULP approach and management among oculoplastic surgeons from Latin America or Spain. Evaluating the current practices of these experts can highlight existing differences and allow for comparison with previous reports^(1,2)^. Additionally, this assessment can aid the selection of the optimal approach for ULP patients. Hence, the present study assessed the current daily practice patterns of members of the Latin American and Spanish societies of Ophthalmic Plastic and Reconstructive Surgery for assessing, diagnosing, and managing ULP.

## METHODS

This study adhered to the tenets of Declaration of Helsinki. The Institutional Ethical Committee of Rio Hortega University Hospital approved this study.

A qualitative web-based survey was conducted in 2020 to assess ULP diagnosis and management among oculoplastic surgeons from Latin America and Spain. The respondents were members of a social network from Latin American countries (a multinational group named OJOPLAST, comprising 403 members; Sociedad Chilena Oculoplastica, Orbita y Cirugía Reconstructiva - SOCHOP, comprising 42 members; and Sociedade Brasileira de Cirurgia Plástica Ocular, comprising 352 members) and Spain (Sociedad Española Cirugía Plástica Ocular - SECPOO, comprising 269 members). Only experts in ULP management were included in this study. If a member belonged to more than one society, only one response was considered.

The sample size calculation indicated that 96 participants were necessary for the survey based on an invited population of 1,066 oculoplastic surgeons, with a 95% confidence level, 10% interval error, and 50% prevalence (applied when the event of interest has an unknown prevalence).

Lime Survey, an open-source software (Lime Survey partners, 2016), was utilized to develop the questionnaire^(3)^. The questionnaire consisted of 40 multiple-choice questions, where all primary questions were mandatory. Some questions required a single response, and other questions required multiple responses. Therefore, the total number of responses could differ per question as some questions allowed for multiple responses.

The original web-based survey is available at the following links:

Portuguese: http://www3.fmb.unesp.br/questionarios/index.php/681395?lang=pt-BR

Spanish: http://www3.fmb.unesp.br/questionarios/index.php/569226?lang=es

A PDF file with an English translation of the questionnaire is available as supplementary material.

In May 2020, an invitation email was sent to oculoplastic surgeons, asking them to participate in this anonymous survey with an end date for participation in July 2020. A total of three reminders were sent at two-week intervals to improve the participation rate.

The survey collected data on surgeons’ demographics and four sections that focused on experience in ptosis management, ULP preoperative evaluation and diagnosis, surgical preferences, postoperative management, and complications. The queries also gathered the participants’ opinions for practical case examples.

ULP grading was defined as follows: mild ULP when the distance from the upper lid margin to the corneal reflex (DMR1) was 3 mm with levator function (LF) >8 mm, and severe ULP was defined as DMR1 <0 and LF 4 mm.

The survey responses were entered into an Excel spreadsheet (Microsoft Corp., Redmond, WA, USA), and occurrence frequency and percentage proportions were calculated. P<0.01 was set to indicate statistical significance.

## RESULTS

The number of respondents was 354 oculoplastic specialists from Spain and South and Central America. The largest proportion of respondents (215/61.1%) was from Brazil. The mean age was similar between the respondents (p=0.0009), with the majority (228/ 64.4%) being between 30 and 50 years old. Most oculoplastic surgeons (68.6% Latin Americans; 71.8% Spanish; p=0.0916) were subspecialists for over 10 years, conducting more than 20 ptosis surgeries annually (55.8% Latin Americans; 45.4% Spanish; p=0.1708).

### Preoperative evaluation

A total of 68.9% of participants reported routinely checking for dry eye preoperatively. The phenylephrine test was reported to be used for both congenital and acquired ptosis (47.4% of respondents) or only acquired ptosis (38.1% of respondents). Phenylephrine at 10% concentration was reported to be used by 56.2% of respondents (mainly Brazilian). There were 48.9% of respondents who reported a common association between ptosis and refractive error.

### Surgical approach

**Figure 1:**
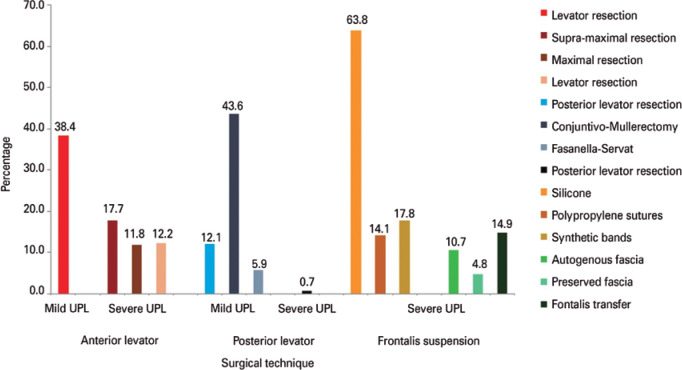
Management of mild or severe upper lid ptosis based on the type of surgical procedure performed from a survey of oculoplastic surgeons considering the possibility of multi-option answers for each question.

The survey responses indicated that multiple options are used to correct ULP (Figure 1). For mild ULP, the majority of surgeons (61.6%) preferred the posterior approach, mainly conjunctival mullerectomy (CMR) (43.6%), followed by anterior levator resection (38.4%). For severe ULP, frontalis techniques were preferred (63.8%), followed by anterior levator resection and supramaximal resection (17.7%) (Figure 1). The frontalis suspension was performed using synthetic materials, mostly silicone tubes (63.8%), braided sutures, such as polypropylene, polyester (14.1%), or synthetic bands, such as polytetrafluoroethylene (17.8%). Biological materials represented the second option, including mostly autogenous fascia (fascia lata or temporal fascia in 10.7%) or homologous preserved fascia (4.8%). The frontalis transfer technique was reported to be another option involving the frontalis muscle (14.9% responders). There were 43.5% of respondents who did not report creating vertical incisions to facilitate frontalis muscle flap displacement.

### Congenital ptosis

Table 1 demonstrates the responses in terms of congenital ptosis management. For severe congenital ptosis, 37.3% of respondents indicated that the age to perform surgery is based on amblyopia development risk. A small proportion of respondents (28.2%) reported using algorithms. Supramaximal resection was reported to be used by 49.4% of respondents for correcting severe congenital ptosis with poor LF. A non-absorbable suture was reported to be preferred to fix the levator to the tarsal plate by 57.1% of respondents. Double palsy (lid ptosis associated with hypotropia) was considered rare by most respondents (68.4%), and the majority (73.7%) reported preferring to initially treat the hypotropia, followed by ULP surgical correction. In Marcus Gunn syndrome with severe synkinesis, 46.6% of the respondents reported performing unilateral surgery and releasing or resecting the levator aponeurosis associated with unilateral frontalis suspension. A total of 51.1% of the respondents reported using the skin-tarsal-skin suture technique to reform the upper lid crease in congenital ptosis. Asymmetry after moderate congenital ptosis surgery was considered the most common complication by 62.7% of the respondents.

**Table 1:** Management of congenital upper lid ptosis from a survey of 354 oculoplastic surgeons considering one option answer for each question

**Question**	**Answer n (%)**	**Answer n (%)**	**Answer n (%)**	**Other n (%)**
Age for surgery in severe congenital ptosis	As soon as diagnosed: 89^*^(25.1)^**^	Over 1 year old: 44 (12.4)	Depends on the risk of amblyopia: 132 (37.3)	89 (25.1)
Use of algorithms	Yes: 100 (28.2)	No: 206 (58.2)		48 (13.6)
Supramaximal resection in severe ptosis	Yes: 175 (49.4)	No: 102 (28.8)		77 (21.7)
Preferred suture type	Absorbable: 100 (28.2)	Non-absorbable: 202 (57.1)	-	52 (14.7)
Double palsy	Frequent: 35 (9.9)	Rare: 242 (68.4)	Underdiagnosed: 29 (8.2)	48 (13.5)
In double paralysis, the order to do surgery must be:	First superior rectus surgery: 261 (73.7)	Superior rectus and levator concomitant surgery: 19 (5.4)	First levator surgery: 27 (7.6)	47 (13.3)
Marcus-Gun syndrome with important unilateral synkinesis	Bilateral levator desinsertion/ resection and frontalis suspension: 73 (20.6)	Unilateral levator desinserction or resection and frontalis suspension: 165 (46.6)		116 (32.8)
To redo the upper lid fold	Skin-tarsus-skin suture: 181 (51.1)	Orbicularis-tarsus then skin: 88 (24.8)		85 (24.0)
Asymmetry after congenital ptosis repair	Common: 222 (62.7)	Rare: 45 (12.7)		87 (24.6)

* = Number of answers;

** ()= Frequency of answer according to the total number of responses (= 354).

### Involutional ptosis

Table 2 shows the responses for involutional ptosis. Considering that involutional ULP frequently presents with concomitant dermatochalasis, levator reinsertion using an anterior approach was favored by 63.3% of respondents when the correction of dermatochalasis and ptosis are warranted. Most respondents (67.8%) reported preferring CRM for mild involutional UPL with good LF and a positive phenylephrine test. Algorithms were used by 58.7% of respondents to plan CRM in involutional ptosis. A non-absorbable suture was the most used material (44.3% of respondents) for CMR. During white line advancement, 47.4% of respondents reported adjusting the height of the lids on the table. In progressive myopathies with negative Bell’s phenomenon and good frontalis muscle function, undercorrection of the frontalis suspension represented the choice for almost half of the respondents (47.7%). Other techniques included levator muscle hyporesection (11%), frontalis linkage/connection (8.5%), flap frontal (5.1%), tarsal switch (3.4%), and other techniques (21.7%). If the frontal muscle is paralytic, frontalis suspension undercorrection was reported to remain the preference (29.4%), followed by undercorrection of the levator resection (23.4%), tarsal switch (16.7%), frontalis linkage/connection (5.6%), frontal flap (2.8%), and other techniques (22%).

**Table 2:** Management of Involutional ptosis from a survey of 354 oculoplastic surgeons considering one option answer for each question

**Question**	**Answer n (%)**	**Answer n (%)**	**Answer n (%)**	**Other n (%)**
Involutional ptosis with good levator function associated with dermochalasis can have the dermochalasis surgery associated to:	Anterior approach to reinsert the levator: 224^*^ (63.3)^**^	Posterior approach to correct ptosis (White line advancement): 31 (8.7)	Posterior approach to correct ptosis (CMR): 28 (7.9)	71(20.1)
Mild ptosis and positive phenylephrine test can be corrected using CMR	Yes = 240 (67.8)	No = 53 (15)		61 (17.2)
Algorithms to CMR in involutional ptosis	Yes = 208(58.7)	No = 84 (23.7)		62(17.5)
Suture thread for CMR	Absorbable: 135 (38.1)	Non-absorbable: 157 (44.3)		62(17.5)
Redo the upper lid fold in involutional ptosis	Yes: 145 (41)	No: 152 (42.9)		57(16.1)
Asymmetry in unilateral involutional ptosis	Common: 82 (23.2)	Rare: 185 (52.2)		87(24.6)

* = Number of answers;

*** () = Frequency of answer according to the total number of responses (= 354); CMR, conjuntivomullerectomy

### Postoperative management

Most of the respondents (65%) reported usually prescribing topical lubricants postoperatively, irrespective of the surgical technique. The Frost suture was reported to be applied after frontalis suspension by 41.2% of the respondents and generally removed after 3 days (30.5%). If warranted, revision surgery was reported to be performed three to six months after the initial procedure by 38.1% of respondents, but 14.7% reported waiting one month, and 11.3% reported performing revision surgery one week postoperatively.

### Complications

**Figure 2:**
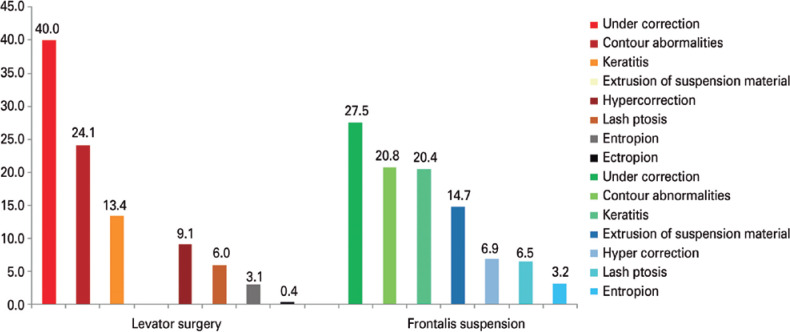
Complications after ptosis repair according to the type of procedure considering multi-option answers for each question.

Figure 2 demonstrates the postoperative complications for severe ptosis. Undercorrection was reported to be the most common complication for aponeurosis resection (40%) or frontalis suspension technique (27.5%).

## DISCUSSION

This web-based survey evaluated ULP diagnosis and management among expert oculoplastic surgeons in Latin America and Spain, highlighting various methods for ULP preoperative evaluation, preferred surgical approaches, management, and main postoperative complications.

The number of surgeons (354 respondents) participating in our survey represents the entire population, as sample size calculations indicated that 96 respondents would be adequate, reinforcing the results of this survey. ASOPRS study sent 552 e-mail requests to the members, and 208 surgeons completed the survey^(1)^. The British study sent 122 e-mail requests, and 53 surgeons participated^(2)^.

Almost half of the surgeons in our survey were well-experienced at managing ULP and performed many surgical procedures (47.7% >20 ULP surgeries/year), similar to the majority of surgeons in the ASOPRS study (92.4% >20 ULP/year)^(1)^.

Almost 2/3 of the respondents were vigilant during postoperative dry eye assessment, which concurs with the ASOPRS study outcomes^(1)^. However, there were no reports of dry eye after anterior levator muscle resection for ULP treatment. This outcome challenges the classic concept that an increase in the palpebral fissure after ptosis repair or blepharoplasty can exacerbate dry eye^(1)^. However, the CMR technique carries the accompanying risk of accessory lacrimal gland removal, thereby decreasing postoperative ocular lubrication^(4-7)^. We believe that a comparative study is warranted to evaluate the subjective and objective parameters of the lacrimal film, comparing the anterior and the posterior approaches for ptosis surgery.

The phenylephrine test was reported to be used to diagnose congenital or acquired ptosis by approximately half of the surgeons (47.4%). This test is commonly used in ptosis patients to identify whether the posterior surgical approach is appropriate^(8)^. Most of our respondents reported using 10% topical phenylephrine, probably due to the lack of a commercial preparation of 2.5% phenylephrine. However, numerous ASOPRS members^(1)^ and BOPSS^(2)^ use 2.5% topical phenylephrine to mitigate potential side effects^(9)^.

Although half of the respondents considered that an association between ptosis and refractive errors was common, the literature indicates that only 16.7% of ULP has been associated with refractive error, anisometropia, strabismus, and amblyopia^(10,11)^. Additionally, severe astigmatism and a change in the astigmatic axis can occur after ptosis surgery^(12)^. Therefore, early detection and timely correction of refractive errors, especially astigmatism, are fundamental to prevent amblyopia.

Blepharoptosis repair is a complex procedure with a variety of surgical approaches. However, there have been no randomized, prospective, controlled comparative studies on ULP surgical techniques^(13)^. The surgical approach is mainly based on ptosis type and surgeon training^(14,15)^. Our survey indicated that most surgeons selected to perform CMR for a posterior approach to correct mild ULP. For mild involutional ptosis, the BPOSS responses indicated that a posterior approach was preferred if the phenylephrine test improved lid height by more than 2 mm; conversely, in cases with less than 2-mm improvement, an anterior approach was favored^(2)^.

Severe ULP correction usually involves various surgical procedures, grouped as anterior or posterior levator approaches and the frontalis muscle techniques. Frontalis suspension was reported to be the preferred technique, followed by the anterior levator approach, mostly supramaximal resection for severe ptosis correction. A notably high proportion of ASOPRS members reported preferring posterior approaches for moderate and severe cases^(1)^.

There are many materials and several technique variations for frontalis suspension. The respondents in the current survey preferred silicone tubes, which corresponds to the outcomes of the ASOPRS survey^(1)^. Biological material, such as autologous fascia, is less frequently used, probably due to greater morbidity and the need for two surgical sites^(1)^.

The method for frontalis muscle mobilization is critical in frontalis muscle advancement. Almost half of the respondents in our survey did not report using vertical incisions to facilitate frontalis muscle flap displacement. Recently, an L-shaped design for the muscle flap was suggested based on the lateral motor innervation of this muscle^(16)^. Similar to a previous study^(17)^, the responses of the current survey indicated that non-absorbable thread was the most frequently used suture for fixing the frontalis muscle to the tarsal plate.

In the current study, alleviating amblyopia risk was the main reason for ULP surgery in children. Previous studies reported an association between amblyopia and severe ULP in 20%^(18)^, 23.9%^(10)^, and 34.2%^(19)^ of children, which was related to anisometropia or stimulus deprivation^(19)^. A small percentage of the respondents in the current study preferred waiting to perform ptosis surgery in children older than one year, probably because early diagnosis and timely treatment of patients with congenital ptosis are essential to prevent amblyopia.

The exact amount of muscle resection to repair ULP is difficult to estimate^(20)^, but most respondents in our study did not report using algorithms, instead opting to identify lid position during intraoperative evaluation^(21)^. Similar to a previous study^(22)^ on congenital ptosis with poor LF, management with supramaximal levator resection was preferred by over half the respondents in the current study.

Consistent with a previous study^(23)^, most respondents in the current study preferred a non-absorbable suture for affixing the levator to the tarsal plate. However, absorbable sutures remain a good option^(22)^.

The technique preferred for upper lid crease reformation is the skin-tarsal-skin suture technique, but approximately 1/3 of our respondents reported initially using the orbicularis-tarsal suture and then skin-to-skin suture. Often, surgical repair improves the lid crease even without lid crease reformation in cases of involutional ptosis^(24)^.

Double palsy was considered rare by the majority of our surgeons, who usually correct strabismus first, followed by ULP^(25)^. However, simultaneous surgery for ptosis and coexisting strabismus can be effective, shortening the treatment period^(26)^.

Almost half of the surgeons in our study preferred using unilateral release or resection of the levator muscle with unilateral frontalis suspension to treat unilateral Marcus Gunn syndrome with severe synkinesis, which is similar to the outcomes of a previous study^(27)^. Thus, bilateral surgery, even in unilateral cases, is advocated for^(28)^, but parents usually do not consent.

Involutional ptosis is often associated with dermatochalasis, and the respondents in the current study and ASOPRS members preferred an anterior approach levator surgery to correct ptosis^(1)^. However, the posterior approach (CRM or white line advancement) can yield excellent results.

The responses from the current survey indicated that CRM is the technique of choice for mild ULP with good LF and a positive phenylephrine test. This outcome is similar to that of the BOPSS survey^(2)^. The amount of posterior resection in CRM can be determined by preo­perative ptosis quantification, response to the phenylephrine test, and existing algorithms^(1,29-32)^. However, the preferred adjustment is determined by evaluating the palpebral height on the table when using white line advancement^(33)^. Non-absorbable sutures are preferred for CRM; however, absorbable sutures are also appropriate^(24,30,32)^.

For challenging cases, such as progressive myopathic ptosis with negative Bell’s phenomenon and/or paralytic frontalis muscle function, the respondents in the current study reported using undercorrected frontalis suspension to protect the cornea, which concurs with the outcomes of a previous study^(34)^. Other options for these cases include the tarsal switch technique^(35,36)^ and frontalis linkage^(37)^.

Postoperatively, artificial tears and the Frost suture are commonly used, generally for 3 days or based on the surgeon’s preference and surgical technique^(38)^. After supramaximal levator resection, the Frost suture can be maintained during bedtime and intermittently during the day in the first postoperative week^(22)^.

The overall revision rate is 8.7% for ptosis repair via posterior or anterior approach^(39)^. Lid position after ptosis repair stabilizes in six weeks^(40)^. For revision surgery, the respondents in the current study generally waited three to six months or from the 1^st^ to the 3^rd^ month (based on patient concerns) and, in some cases, one or two weeks postoperatively^(39,41)^. A previous study suggested that early postoperative adjustment can decrease the interval to achieve the final result^(42)^.

Complications after severe ptosis surgery depend on the surgical technique. In our survey, aponeurotic procedures or frontalis suspension were the most likely to result in an undercorrection, but variations in the technique might have influenced the outcome^(43,44)^.

Contour abnormalities in our study are similar to those reported by a previous publication^(22)^, including levator resection or frontalis suspension, and these abnormalities are commonly observed after supramaximal levator resection.

There are some limitations to this study. Selec­tion bias can be an inherent flaw of surveys. However, the homogenous distribution of responses ensured that our participants’ opinions did not differ from that of non-par­ticipants. A limitation of our survey was that it did not identify which society the participants belonged to, but the intention of the study was not to compare the responses between members of different societies. Additionally, the greater proportion of respondents was Brazilian, probably due to the larger population of ophthalmologists in Brazil. Lastly, less common causes of ULP requiring other approaches were not examined. Therefore, interpretation should be limited to commonly performed techniques, although less commonly used techniques may yield similar surgical outcomes.

In conclusion, this study highlights the methods for ULP diagnosis and treatment and postoperative complications based on ULP type and technique used by members of the Latin America and the Spanish Oculoplastic subspecialty. The outcomes of this study can specifically help new surgeons during the challenges of managing ULP.

## References

[1] Aakalu VK, Setabutr P (2011). Current ptosis management: a national survey of ASOPRS
members. Ophthal Plast Reconstr Surg.

[2] Mota PM, Norris JH (2016). Review on surgical management of ptosis and the use of
phenylephrine: A national survey of British Oculoplastic Surgery Society
(BOPSS) UK Consultants. Orbit.

[3] Lime Survey Partners (2016). LimeSurveyPartners.

[4] Zloto O, Matani A, Prat D, Leshno A, Ben Simon G (2020). The Effect of a ptosis procedure compared to an upper
blepharoplasty on dry eye syndrome. Am J Ophthalmol.

[5] Bautista SA, Wladis EJ, Schultze RL (2018). Quantitative assessment of dry eye parameters after
müller’s muscle-conjunctival resection. Ophthal Plast Reconstr Surg.

[6] Bodian M (1989). Does conjunctival resection in ptosis surgery lead to dry-eye
syndrome?. Ann Ophthalmol.

[7] Rymer BL, Marinho DR, Cagliari C, Marafon SB, Procianoy F (2017). Effects of Muller’s muscle-conjunctival resection for ptosis on
ocular surface scores and dry eye symptoms. Orbit.

[8] Barsegian A, Botwinick A, Reddy HS (2018). The phenylephrine test revisited. Ophthal Plast Reconstr Surg.

[9] Glatt HJ, Fett DR, Putterman AM (1990). Comparison of 2.5% and 10% phenylephrine in the elevation of
upper eyelids with ptosis. Ophthalmic Surg.

[10] Srinagesh V, Simon JW, Meyer DR, Zobal-Ratner J (2011). The association of refractive error, strabismus, and amblyopia
with congenital ptosis. J AAPOS.

[11] Thapa R (2010). Refractive error, strabismus and amblyopia in congenital
ptosis. JNMA J Nepal Med Assoc.

[12] Paik JS, Kim SA, Park SH, Yang SW (2016). Refractive error characteristics in patients with congenital
blepharoptosis before and after ptosis repair surgery. BMC Ophthalmol.

[13] Chang S, Lehrman C, Itani K, Rohrich RJ (2012). A systematic review of comparison of upper eyelid involutional
ptosis repair techniques: efficacy and complication rates. Plast Reconstr Surg.

[14] McCord CJ, Codner M, Hester TJ (1995). Eyelid Surgery: Principles and Techniques.

[15] Finsterer J (2003). Ptosis: causes, presentation, and management. Aesthetic Plast Surg.

[16] Cruz AA, Akaishi AP (2018). Frontalis-Orbicularis Muscle Advancement for Correction of Upper
Eyelid Ptosis: A Systematic Literature Review. Ophthal Plast Reconstr Surg.

[17] Medel R, Alonso T, Giralt J, Torres J, González-Candial M, García-Arumí J (2006). Frontalis muscle flap advancement with a pulley in the levator
aponeurosis in patients with complete ptosis and deep-set
eyes. Ophthal Plast Reconstr Surg.

[18] Anderson RL, Baumgartner SA (1980). Amblyopia in ptosis. Arch Ophthalmol.

[19] Kasaee A, Yazdani-Abyaneh A, Tabatabaie SZ, Jafari AK, Ameri A, Eshraghi B (2010). Assessing amblyogenic factors in 100 patients with congenital
ptosis. Int J Ophthalmol.

[20] Mauriello JA, Wagner RS, Caputo AR, Natale B, Lister M (1986). Treatment of congenital ptosis by maximal levator
resection. Ophthalmology.

[21] Harvey DJ, Iamphongsai S, Gosain AK (2010). Unilateral congenital blepharoptosis repair by anterior levator
advancement and resection: an educational review. Plast Reconstr Surg.

[22] Cruz AA, Akaishi PM, Mendonça AK, Bernadini F, Devoto M, Garcia DM (2014). Supramaximal levator resection for unilateral congenital ptosis:
cosmetic and functional results. Ophthal Plast Reconstr Surg.

[23] Repp DJ, Rubinstein TJ, Sires BS (2017). Role of algorithm-based levator aponeurectomy in small-incision
external ptosis surgery for involutional ptosis. JAMA Facial Plast Surg.

[24] Couch SM (2016). Correction of Eyelid Crease Asymmetry and Ptosis. Facial Plast Surg Clin North Am.

[25] Weaver DT (2018). Current management of childhood ptosis. Curr Opin Ophthalmol.

[26] Zhou F, Ouyang M, Ma D, Liu G, Cheng H (2017). Combined surgery for simultaneous treatment of congenital ptosis
and coexisting strabismus. J Pediatr Ophthalmol Strabismus.

[27] Bowyer JD, Sullivan TJ (2004). Management of Marcus Gunn jaw winking synkinesis. Ophthal Plast Reconstr Surg.

[28] Khwarg SI, Tarbet KJ, Dortzbach RK, Lucarelli MJ (1999). Management of moderate-to-severe Marcus-Gunn jaw-winking
ptosis. Ophthalmology.

[29] Putterman AM (1985). Müllers muscle-conjunctival resection ptosis
procedure. Aust N Z J Ophthalmol.

[30] Weinstein GS, Buerger GF Jr (1982). Modification of the Müller’s muscle-conjunctival resection
operation for blepharoptosis. Am J Ophthalmol.

[31] Dresner SC (1991). Further modifications of the Müller’s muscle-conjunctival
resection procedure for blepharoptosis. Ophthal Plast Reconstr Surg.

[32] Perry JD, Kadakia A, Foster JA (2002). A new algorithm for ptosis repair using conjunctival
Müllerectomy with or without tarsectomy. Ophthal Plast Reconstr Surg.

[33] Al-Abbadi Z, Sagili S, Malhotra R (2014). Outcomes of posterior-approach ‘levatorpexy’ in congenital ptosis
repair. Br J Ophthalmol.

[34] Bernardini FP, de Conciliis C, Devoto MH (2002). Frontalis suspension sling using a silicone rod in patients
affected by myogenic blepharoptosis. Orbit.

[35] Massry GG, Hornblass A, Rubin P, Holds JB (1999). Tarsal switch procedure for the surgical rehabilitation of the
eyelid and socket deficiencies of the anophthalmic socket. Ophthal Plast Reconstr Surg.

[36] Meneghim RL, Ferraz LB, Galindo-Ferreiro A, Khandekar R, Sanchez-Tocino H, Schellini S (2018). Tarsal switch using an anterior approach to correct severe
ptosis. Arch Plast Surg.

[37] Diniz SB, Akaishi PM, Cruz AA (2020). Frontalis Linkage Without Intraoperative Eyelid Elevation for the
Management of Myopathic Ptosis. Ophthal Plast Reconstr Surg.

[38] Desciak EB, Eliezri YD (2003). Surgical Pearl: temporary suspension suture (Frost suture) to
help prevent ectropion after infraorbital reconstruction. J Am Acad Dermatol.

[39] Chou E, Liu J, Seaworth C, Furst M, Amato MM, Blaydon SM, Durairaj VD, Nakra T, Shore JW (2018). Comparison of revision rates of anterior- and posterior-approach
ptosis surgery: a retrospective review of 1519 cases. Ophthalmic Plast Reconstr Surg.

[40] Tucker SM, Verhulst SJ (1999). Stabilization of eyelid height after aponeurotic ptosis
repair. Ophthalmology.

[41] Shore JW, Bergin DJ, Garrett SN (1990). Results of blepharoptosis surgery with early postoperative
adjustment. Ophthalmology.

[42] Dortzbach RK, Kronish JW (1993). Early revision in the office for adults after unsatisfactory
blepharoptosis correction. Am J Ophthalmol.

[43] Pak J, Shields M, Putterman AM (2006). Superior tarsectomy augments super-maximum levator resection in
correction of severe blepha­roptosis with poor levator
function. Ophthalmology.

[44] Galindo-Ferreiro A, Akaishi P, Hanafi S, Khandekar R, Galvez-Ruiz A, Schellini S (2017). Outcomes of two surgical techniques using silicone rod for
frontalis sling suspension to treat severe ptosis. J Pediatr Ophthalmol Strabismus.

